# Postcardiac injury syndrome after cardiac implantable electronic device implantation

**DOI:** 10.1007/s00059-020-04910-6

**Published:** 2020-03-13

**Authors:** Kevin Filbey, Farbod Sedaghat-Hamedani, Elham Kayvanpour, Panagiotis Xynogalos, Daniel Scherer, Benjamin Meder, Hugo A. Katus, Edgar Zitron

**Affiliations:** 1 Department of Medicine III, Universitätsklinikum Heidelberg, INF 410, 69120 Heidelberg, Germany; 2 DZHK (German Centre for Cardiovascular Research), Heidelberg, Germany

**Keywords:** Cardiac resynchronization therapy devices, Pericarditis, Pericardial effusion, Dilated cardiomyopathy, Right atrial lead implantation, Kardiale Resynchronisationstherapiegeräte, Perikarditis, Perikarderguss, Dilatative Kardiomyopathie, Rechtsatriale Elektrodenimplantation

## Abstract

**Background:**

Postcardiac injury syndrome (PCIS) is an inflammatory complication that derives from injury to the epicardium, myocardium, or endocardium. It occurs after trauma, myocardial infarction, percutaneous coronary intervention, cardiac surgery, intracardiac ablation, and implantation of cardiac implantable electronic device (CIED). In this study we assessed the incidence of PCIS after CIED implantation and its possible risk factors.

**Material and methods:**

All patients who received CIED implantation at Heidelberg University Hospital between 2000 and 2014 were evaluated (*n* = 4989 patients). Clinical data including age, sex, underlying cardiac disease, type of implanted CIED, location of electrode implantation, clinical symptoms, time of symptom onset of PCIS, therapy, and outcome were extracted and analyzed.

**Results:**

We identified 19 cases of PCIS in 4989 patients, yielding an incidence of 0.38%. The age of patients with PCIS ranged from 39 to 86 years. Dilated cardiomyopathy (DCM) as underlying cardiac disease and right atrial (RA) lead implantation had a significant association with occurrence of PCIS (*p* = 0.045 in DCM and *p* < 0.001 in RA lead implantation). Dyspnea, chest pain, dry cough, and fever were the most frequently reported symptoms in patients with PCIS. Pericardial and pleura effusion as well as elevated C‑reactive protein (CRP), increased erythrocyte sedimentation rate (ESR), and leukocytosis were the most common findings.

**Conclusion:**

To the best of our knowledge, this is the largest cohort evaluating the incidence of PCIS after CIED implantation. The data show that PCIS is a rare complication after CIED implantation and occurs more frequently in patients with DCM and those with RA lead implantation. Although rare and mostly benign, PCIS can lead to potentially lethal complications and physicians must be aware of its symptoms.

**Electronic supplementary material:**

The online version of this article (10.1007/s00059-020-04910-6) contains supplementary material, which is available to authorized users.

## Abstract (de)

**Hintergrund:**

Das Syndrom infolge Herzschädigung („post-cardiac injury syndrome“, PCIS) stellt eine inflammatorische Komplikation dar, die durch eine Schädigung des Epikards, Myokards oder Endokards hervorgerufen wird. Es kann nach einem Trauma, Herzinfarkt, einer perkutanen Koronarintervention, einem herzchirurgischen Eingriff, intrakardialer Ablation und Implantation eines kardialen implantierbaren elektronischen Systems („cardiac implantable electronic device“, CIED) auftreten. In der vorliegenden Studie wurden die Inzidenz des PCIS nach CIED-Implantation und mögliche Risikofaktoren dafür ermittelt.

**Material und Methoden:**

Sämtliche Patienten, die am Universitätsklinikum Heidelberg zwischen 2000 und 2014 ein CIED implantiert bekamen, wurden in die Auswertung einbezogen (*n* = 4989 Patienten). Klinische Daten zu Alter, Geschlecht, zugrunde liegender Herzerkrankung, Art des implantierten CIED, Ort der Elektrodenimplantation, klinischen Symptomen, Zeitpunkt des PCIS-Symptombeginns, Therapie und Ergebnis wurden erfasst und ausgewertet.

**Ergebnisse:**

Die Autoren fanden 19 Fälle von of PCIS unter 4989 Patienten, was eine Inzidenz von 0,38 % ergab. Das Alter der Patienten mit PCIS lag zwischen 39 und 86 Jahren. Eine dilatative Kardiomyopathie (DCM) als zugrunde liegende Herzerkrankung und die rechtsatriale (RA-)Elektrodenimplantation wiesen einen signifikanten Zusammenhang mit dem Auftreten eines PCIS auf (*p* = 0,045 bei DCM und *p* < 0,001 bei RA-Elektrodenimplantation). Dyspnoe, Brustschmerzen, trockener Husten und Fieber waren die am häufigsten angegebenen Symptome bei Patienten mit PCIS. Ein Perikard- und Pleuraerguss sowie eine Erhöhung des Werts für C‑reaktives Protein (CRP), erhöhte Blutsenkungsgeschwindigkeit (BSG) und Leukozytose stellten die häufigsten Befunde dar.

**Schlussfolgerung:**

Nach Wissen der Autoren handelt es sich hier um die größte Kohorte, in der die Inzidenz des PCIS nach CIED-Implantation ermittelt wurde. Die Daten zeigen, dass PCIS eine seltene Komplikation nach CIED-Implantation ist und häufiger bei Patienten mit DCM und mit RA-Elektrodenimplantation auftritt. Auch wenn es selten und zumeist benigne ist, kann das PCIS zu potenziell tödlichen Komplikationen führen, Ärzte müssen sich daher seiner Symptome bewusst sein.

Postcardiac injury syndrome (PCIS) is an inflammatory response to epicardial, myocardial, or endocardial injuries. It can follow cardiac surgery, myocardial infarction, trauma, intracardiac ablation, percutaneous coronary intervention, or implantation of cardiac implantable electronic device (CIED; [1–4]). The underlying pathogenesis is thought to be an autoimmune reaction directed toward the contractile cardiac proteins, which are exposed after cardiac injury. This leads to an inflammation of the pericardium that manifests itself in pericardial effusion (PE; [1, 5]).

The most common cause of PCIS is cardiac surgery, with an incidence of approximately 15–30% [6]. The severity of the autoimmune reaction seems to correlate with the level of antiheart antibodies (AHAs; [5, 7]). It is unclear whether elevated levels of AHAs are the actual cause of PCIS or only an epiphenomenon [8]. A viral origin is also possible [9]. The exact incidence of PCIS after CIED implantation is unclear and has been estimated to be approximately 0.2–5% and might be related to implantation technique, lead tip position, and design [1, 10–13]. The incidence of PCIS in patients receiving CIED implantation via active fixation has been shown to be significantly higher than those with passive fixation [12]. Active fixation not only creates a greater injury, it also makes micro-perforation of the myocardium more likely. This causes a greater release of cardiac proteins and therefore a more severe immune response [10, 12, 14].

Being a rare complication, the diagnosis of PCIS remains difficult. Clinical symptoms and signs such as dyspnea, thoracic pain, fever, pericardial friction, and PE, in addition to elevated inflammatory parameters (erythrocyte sedimentation rate [ESR], C‑reactive protein (CRP), and leukocytes), are the most widely reported symptoms and findings in PCIS [15–17]. In this large retrospective study, we evaluated the incidence and possible risk factors leading to PCIS after CIED implantation via active lead fixation.

## Material and methods

This retrospective study was performed after approval of the institutional ethics committee of the University of Heidelberg and in accordance with national ethical standards. We performed a single-center retrospective study and included all patients who received a CIED at the University Hospital in Heidelberg between 2000 and 2014. From a total number of 5305 patients, 316 were excluded owing to insufficient documentation. This left a remaining number of 4989 patients for further analysis. Clinical data were extracted on age, sex, underlying cardiac disease, type of CIED (cardiac resynchronization therapy [CRT]; pacemaker [PM]; implantable cardioverter defibrillator [ICD]), location of lead implantation (right atrium [RA], right ventricle [RV], coronary sinus [CS]), time of symptom onset of PCIS, laboratory parameters (ESR, CRP, leukocyte count), clinical signs and symptoms, therapy, and outcome. The observation period started on the day of implantation and ended after 6 months. The diagnosis of PCIS was made based on the development of PE following CIED implantation with clinical signs of PCIS. These included accompanying PE and elevation of inflammatory biomarkers while no other plausible explanation for the PE was present. The chosen criteria were derived from previous studies [1, 4, 13]. Chest X‑ray and echocardiography were performed routinely to exclude any dislocation of electrodes. All patients received active fixation of the implanted lead.

### Statistical analysis

A retrospective analysis for statistical significance was conducted. Parameters that showed statistical significance in univariate analysis were then included in further evaluations using multivariate logistic regression. Survival analyses are shown as Kaplan–Meier curves. Sex, symptoms, and other characteristics of the collective are reported using descriptive analysis.

## Results

### PCIS is a rare complication after CIED implantation

All 4989 patients received CIED implantation via active lead fixation. The average age of patients who received CIED was 68.4 years (±13.3), 70.5% of patients were male. The most common underlying cardiovascular diseases were ischemic cardiomyopathy (ICM, 48.5%) followed by nonischemic dilated cardiomyopathy (DCM, 22.8%). Baseline characteristics of all analyzed patients are listed in Table 1. After retrospective clinical evaluation of all patients, only 0.38% (19 of 4989) developed signs and symptoms of PCIS during the 6‑month follow-up period (Table 2).

**Table 1:** Baseline characteristics of all patients included in the study (*n* = 4989)

*Baseline characteristics*	
Age (mean ± SD, years)	68.40 (±13.3)
Male sex (%)	3516 (70.5%)
*CIED type*	
ICD	1941 (38.9%)
PM	1940 (38.9%)
CRT D/P	1108 (22.2%)
*Cardiac disease*	
ICM	2419 (48.5%)
DCM	1136 (22.8%)
HCM	122 (2.4%)
ARVC	20 (0.4%)
LVNC	13 (0.3%)
Amyloidosis	19 (0.3%)
SSS	285 (5.7%)
Conduction disease	491 (9.8%)
Bradyarrhythmia	266 (5.3%)
Others	218 (4.3%)

*ARVC* arrhythmogenic right ventricular cardiomyopathy, *CIED* cardiac implantable electronic device, *CRT D/P* cardiac resynchronization therapy, *DCM* dilatative cardiomyopathy, *HCM* hypertrophic cardiomyopathy, *ICD* intra cardiac defibrillator, *ICM* ischemic cardiomyopathy, *LVNC* left ventricular noncompaction cardiomyopathy, *PM* pacemaker, *SSS* sick sinus syndrome

**Table 2:** Cases of postcardiac injury syndrome (PCIS) in the patient collective

Patient	Age	Sex	Underlying disease	Reason for implantation	CIED	RA electrode	Therapy	Outcome
1	77	m	ICM	Bradycardia	PM	Yes	Pericardiocentesis	Responding
2	51	m	ICM	Reduced LVEF	CRT‑D	No	None	Responding
3	67	f	HCM	Syncope, primary prophylaxis	ICD	Yes	Pericardiocentesis	Death due to cardiogenic shock
4	78	f	SSS	Bradycardia	PM	Yes	None	Responding
5	57	m	ICM	Reduced LVEF	CRT‑D	Yes	Pericardiocentesis	Responding
6	78	f	Hypertensive heart disease	Bradycardia	PM	Yes	Steroids	Responding
7	79	m	Conduction disease	AVB III°	PM	Yes	Pericardiocentesis	Responding
8	64	f	DCM	Reduced LVEF	CRT‑D	Yes	Steroids	Responding
9	81	f	ICM	VT	ICD	Yes	None	Responding
10	80	m	ICM	Bradycardia	PM	Yes	None	Responding
11	81	m	ICM	AVB III°	PM	Yes	Pericardiocentesis	Responding
12	86	f	DCM	Reduced LVEF	CRT‑D	Yes	None	Responding
13	63	m	DCM	Bradycardia	PM	Yes	NSAID + steroids	Responding
14	43	m	DCM	Reduced LVEF	CRT‑D	Yes	NSAID	Responding
15	39	m	DCM	Reduced LVEF	CRT‑D	Yes	Steroids	Responding
16	81	f	SSS	Bradycardia	PM	Yes	Exchange of electrode	Responding
17	60	f	DCM	Bradycardia	PM	Yes	Steroids	Responding
18	66	m	DCM	Reduced LVEF	CRT‑D	Yes	Steroids	Responding
19	57	f	DCM	Reduced LVEF	CRT‑D	Yes	Pericardiocentesis	Responding

*AVB* atrioventricular block, *AF* atrial fibrillation, *CIED* cardiac implantable electronic device, *CM* cardiomyopathy, *CRT‑D* cardiac resynchronization therapy defibrillator, *f* female, *DCM* dilatative cardiomyopathy, *HCM* hypertrophic cardiomyopathy, *ICD* intra cardiac defibrillator, *ICM* ischemic cardiomyopathy, *LVEF* left ventricular ejection fraction, *m* male, *NSAID* nonsteroidal anti-inflammatory drugs, *PM* pacemaker, *SSS* sick sinus syndrome

### RA lead implantation and DCM are associated with higher incidence of PCIS

**Fig. 1:**
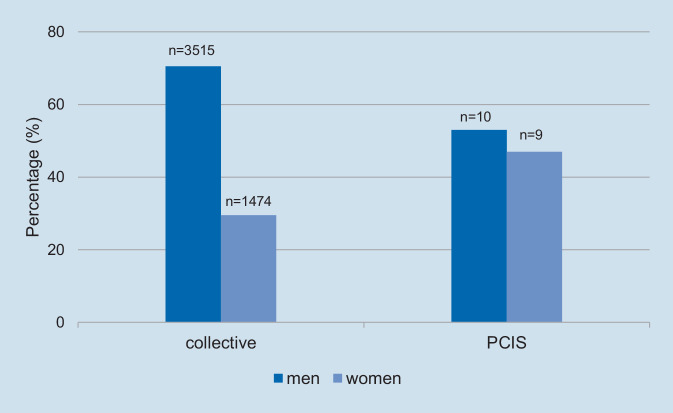
Sex distribution in the whole patient collective and in the postcardiac injury syndrome (*PCIS*) group. The *left bars* show the distribution in the collective and the *right bars* show this in patients with PCIS. No significant difference was apparent (*p* = 0.088)

**Fig. 2:**
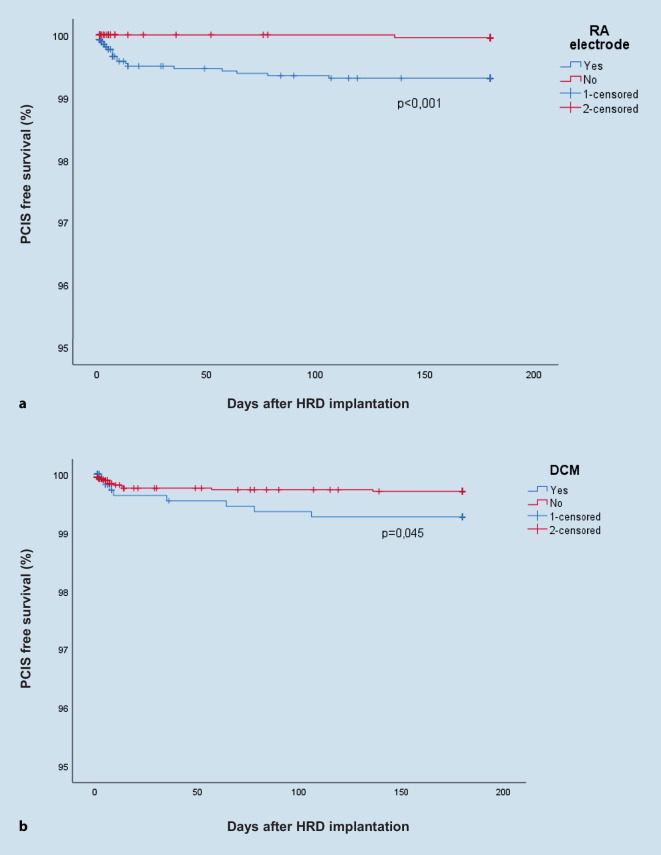
Kaplan–Meier survival analysis based on occurrence of postcardiac injury syndrome (*PCIS*) after cardiac implantable electronic device/heart rhythm device (CIED/HRD) implantation. **a** The incidence of PCIS in patients with right atrial (*RA*) lead implantation is significantly higher (*p* = 0.008; OR = 15.56). **b** The incidence of PCIS in patients with dilated cardiomyopathy (*DCM*) is significantly higher (*p* = 0.045; OR = 2.60)

The average age of patients who developed PCIS was 67.8 years (±13.9) and ten of 19 patients (52.6%) were male. There was no statistical significance for age or sex between all patients and the PCIS group (Fig. 1). However, there was a highly significant correlation between RA lead implantation and the development of PCIS (*p* < 0.001). In addition, DCM as underlying cardiac disease also showed a significant correlation with the development of PCIS (*p* = 0.04). Out of 19 patients, 18 (94.7%) received RA lead implantation and eight out of 19 (42.1%) had been diagnosed with DCM. In a multivariate logistic regression analysis, both of these factors were found to be independent predictors of PCIS development (OR = 15.2; CI 95% 2.1–116.7; *p* = 0.008 for RA lead implantation site and OR = 2.6; CI 95% 1.0–6.5; *p* = 0.04 for DCM). The corresponding Kaplan–Meier curves of the PCIS-free survival analysis are shown in Fig. 2.

### Clinical finding and symptoms in patients with PCIS

The median onset of symptoms was 7 days after implantation, with 1 day being the earliest and 136 days the latest. In 68% of cases (*n* = 13/19), the symptoms manifested within the first 14 days. Elevated levels of CRP and ESR showed a high prevalence in patients with PCIS, with pleural effusion occurring in every second patient. Other symptoms such as dyspnea, chest pain, unproductive cough, and fever were also present. Nausea, edema, and fatigue were less common. Clinical symptoms and laboratory parameters with their corresponding prevalence are listed in Table 3.

**Table 3:** Clinical symptoms and laboratory parameters in PCIS group

Clinical findings	Percentage % (*n*)
Pericardial effusion	100 (19/19)
Elevated ESR	100 (8/8)
Elevated CRP	95 (18/19)
Elevated leukocytes	68 (13/19)
Pleural effusion	50 (9/18)
*Symptoms*	
Dyspnea	53 (10/19)
Chest pain	32 (6/16)
Unproductive cough	26 (5/19)
Fever	26 (5/19)
Fatigue	16 (3/19)
Edema	11 (2/19)
Nausea	5 (1/19)

*PCIS* postcardiac injury syndrome, *ESR* erythrocyte sedimentation Rate, *CRP* C-reactive protein

### Therapy and outcome of PCIS patients

Out of 19 patients, five received only symptomatic therapy owing to mild symptoms. Seven patients (37%) received anti-inflammatory treatment with NSAIDs, steroids, or both. In six cases, pericardiocentesis was performed, and in one patient the lead was replaced. Except for one, all patients showed an improvement of symptoms after therapy without recurrences. This patient received intensive care treatment for sepsis. He developed pericardial effusion, which was treated via pericardial paracentesis. Still, the patient’s condition worsened leading to cardiogenic shock and ultimately cardiac arrest in the form of electromechanical dissociation.

## Discussion

Postcardiac injury syndrome can occur in the setting of injury to the pericardium, epicardium, or myocardium [4]; PCIS after CIED implantation is a rare complication. Its incidence in this study was 0.38%, which was lower than formerly reported [10–13]. Since this is the largest study conducted on this subject to date (4989 patients included), it is likely that the frequency we present reflects the true incidence of PCIS. A further plausible explanation for this finding might be the use of more modern lead designs in our patients (2000–2014), in comparison with Sivakumaran et al. (1991–1999) or Greene et al. (1989–1990) [11, 12, 18]. Additional reasons for varying frequencies might be the lack of clear diagnostic criteria, as well as the fact that mild cases of PCIS are most likely never identified.

Even though the pathomechanism of PCIS remains unclear, studies suggest an autoimmune reaction toward the contractile proteins of the heart to be the most likely cause [1, 5, 7, 19, 20]. This hypothesis is supported by the effectiveness of anti-inflammatory drugs and the findings of elevated levels of AHAs in the serum of PCIS patients. Nevertheless, it is unclear whether AHAs play a primary role in the pathogenesis of PCIS. Hoffman et al. suggest that serum AHAs may be an epiphenomenon rather than a cause of PCIS, since all patients tested negative for AHAs before PCIS onset and they tested positive after only 14 days [8]. There is also evidence showing that the autoimmune reaction might be triggered by viral infection [9, 21]. Moreover, PCIS after CIED implantation may be triggered by direct irritation of the pericardium caused by slightly protruding electrodes [4]. Sex showed no significant effect on PCIS development in this study (*p* = 0.088). Nonetheless, the incidence of PCIS among female patients (0.61%, *n* = 9/1473) was at least twice as high as among male patients (0.28%, *n* = 10/3516). This implies a possible gender-associated risk factor. As expected, PCIS is more likely to develop in patients with lead implantation in the thinner atrial wall, supporting the theory of increased autoimmune reaction due to micro-perforation of the myocardium [10, 12, 14]. Furthermore, DCM as underlying cardiac disease seems to be associated with the development of PCIS and should be considered a possible risk factor. On the one hand, the dilatation and therefore thinning of the myocardium might increase the likelihood of micro-perforation and therefore the risk of PCIS. On the other hand, the activated inflammatory pathways in DCM might play a role in the development of PCIS. The link between inflammation and DCM was first described by Anderson et al. [22]. They showed an abnormal activity of natural killer cells (NKCs) in the majority of DCM patients [22]. Besides, further studies showed high levels of circulating tumor necrosis factor (TNF), interleukine‑6 (IL-6), and IL-18 in patients with end-stage chronic heart failure as well as an analysis of the effect of pro-inflammatory cytokines on left ventricular (LV) remodeling and the progression of heart failure [23–25]. The onset of PCIS is reported from 1 day to 4 months after CIED implantation [4]. The reason for this long time span is unclear. As it is suggested to be an autoimmune reaction, this could happen at any time; however, later onset of symptoms, especially later than 5 months, makes the diagnosis of PCIS less likely.

**Fig. 3:**
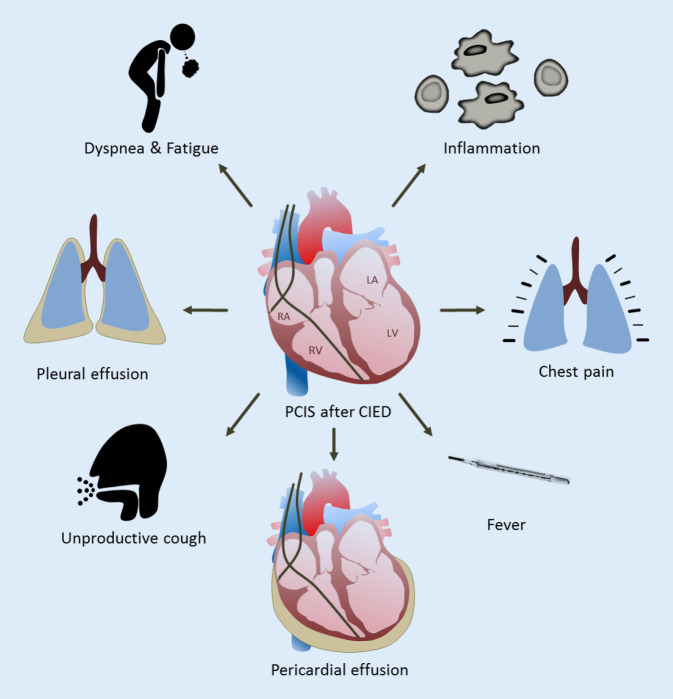
Schematic view of signs and symptoms of postcardiac injury syndrome (*PCIS*). Pericardial effusion, elevation of inflammatory biomarkers, dyspnea, fatigue, fever, cough, and pleural effusion are the most common symptoms and findings in patients with PCIS. *CIED* cardiac implantable electronic device, *LA* left atrium, *LV* left ventricle, *RA* right atrium, *RV* right ventricle

The most important and challenging measures seem to be the correct diagnosis of PCIS and the awareness of its possible lethal outcome. There is no clear consensus regarding the diagnosis of PCIS. In general, the diagnosis is based on comprehensive clinical history-taking, evidence of inflammation, and pericarditis with PE (Fig. 3; [1, 4]). Possible imaging includes transthoracic ultrasound and computed tomography. Bacterial infection can be ruled out with x‑ray, blood and urinary culture, or procalcitonin (PCT). Further, acute diseases such as acute coronary syndrome, pulmonary embolism, or pneumonia must be ruled out first [4]. The treatment of PCIS is based on anti-inflammatory therapy [4, 8, 26]. The recommended first-line therapy with NSAIDs and steroids showed a beneficial effect on our patients. Similarly, positive therapeutic results were observed after pericardiocentesis in hemodynamically compromised patients. In some cases, clinical symptoms and PE regressed spontaneously. It seems that the need for therapy might be limited to moderate and severe cases of PCIS, the latter needing more invasive treatment. In general, careful observation of such patients is strongly recommended. Most cases seem to occur during the first 2 weeks after CIED implantation; nonetheless, later onsets must be considered. In persisting, worsening, or recurring cases of PCIS, patients should be treated with low-dose steroids (0.2–0.5 mg/kg/day; [26]). Hemodynamically compromised patients seem to benefit from pericardiocentesis.

## Conclusion

Postcardiac injury syndrome remains a rare, yet potentially lethal complication of cardiac implantable electronic device implantation. The incidence after active lead fixation seems to be lower than previously described (0.38%). PCIS should be considered particularly in patients with right atrial lead active fixation and dilated cardiomyopathy as underlying cardiac disease. Pericarditis and elevation of inflammatory biomarkers are useful hints in the diagnosis of PCIS, especially in combination with other clinical parameters such as dyspnea, fever, cough, and pleural effusion. The suggested first-line therapy with nonsteroidal anti-inflammatory drugs and steroids showed beneficial results and is recommended. In hemodynamically compromised patients, pericardiocentesis should be considered.

## Caption Electronic Supplementary Material

Supplementary Table 1: pacing parameters of PCIS case
