# Supplementary material for: Postcardiac injury syndrome after cardiac implantable electronic device implantation
Source: Herz. 2020 Mar 13;45(7):696–702. doi: 10.1007/s00059-020-04910-6 (PMC7581580; doi:10.1007/s00059-020-04910-6)
Supplement: Supplementary file 1 — Supplementary Table 1: pacing parameters of PCIS case [file 59_2020_4910_MOESM1_ESM.docx]

|  | **Intra-operative** | | | | | | **PCIS** | | | | | |
| --- | --- | --- | --- | --- | --- | --- | --- | --- | --- | --- | --- | --- |
|  | **Right atrium** | | | **Right ventricle** | | | **Right atrium** | | | **Right ventricle** | | |
| **Patients** | **impedance (Ω)** | **Threshold**  **(V/ms)** | **Sensing**  **(mv)** | **impedance (Ω)** | **Threshold**  **(V/ms)** | **Sensing**  **(mv)** | **impedance (Ω)** | **Threshold**  **(V/ms)** | **Sensing**  **(mv)** | **impedance (Ω)** | **Threshold**  **(V/ms)** | **Sensing**  **(mv)** |
| 1 | 717 | 1/0,4 | 2,8 | 773 | 0,5/0,4 | 16,3 | 489 | 1V /0,4ms | 2,8 | 452 | 0,7/0,4 | 16,4 |
| 2 | 459 | 0,5/0,5 | 3,7 | 435 | 0,5/0,5 | 14,6 | 603 | 1,5/0,5 | 6,5 | 709 | 0,5/0,5 | 16,8 |
| 3 | 484 | 1/0,5 | 5,5 | 766 | 0,7/0,5 | 21,4 | 347 | 0,4/0,5 | 4,3 | 334 | 1,8/0,5 | NA |
| 4 | 520 | 1,1/0,5 | 3,2 | 1137 | 0,4/0,5 | 12,1 | 446 | 0,8/0,4 | 2,9 | 1120 | 0,7/0,4 | 21,9 |
| 5 | 508 | 0,9V/0,5 | 2,3 | 622 | 0,4/0,5 | 20 | 478 | 0,7/0,5 | 7,4 | 467 | 0,5/0,5 | 21,4 |
| 6 | 511 | 0,5/0,5 | 2,8 | 788 | 0,9/0,5 | 9,6 | 318 | NA/AF | 2,9 | 582 | 0,75/0,35 | 14,2 |
| 7 | NA | NA | NA | 692 | 0,5/0,35 | 16,2 | NA | NA | NA | 598 | 1,25/0,35 | 16,2 |
| 8 | 395 | 0,8/0,5 | 4,6 | 587 | 0,7/0,5 | 21,1 | 411 | 0,3/0,5 | 3,1 | 338 | 1,0/0,5 | 8,6 |
| 9 | 470 | 1,1/0,5 | 1,6 | 775 | 1,3/0,5 | 9,6 | 470 | 1,0/0,4 | 2,9 | 486 | 0,7/0,5 | 16,8 |
| 10 | 769 | 1,3/0,5 | 2,1 | 932 | 0,4/0,5 | 16,7 | NA | NA | NA | NA | NA | NA |
| 11 | 470 | 0,6/0,5 | 4,5 | 630 | 0,5/0,5 | 12,5 | 472 | 1,0/0,4 | 5,6 | 599 | 0,5/0,4 | NA |
| 12 | 360 | 1,2/0,5 | 2,3 | 580 | 0,5/0,5 | 11,4 | 390 | NA/AF | 0,8 | 590 | 0,75/0,5 | 11,4 |
| 13 | 479 | 0,9/0,5 | 1,6 | 853 | 0,7/0,5 | 8,2 | 523 | NA/AF | 3,6 | 586 | 0,75/0,4 | 12 |
| 14 | 421 | 1,3/0,5 | 3,5 | 433 | 0,5/0,5 | 7,5 | 440 | 0,5/0,5 | 2,5 | 430 | 0,5/0,5 | 11,4 |
| 15 | 592 | 0,6/0,5 | 6 | 627 | 0,6/0,5 | 10 | 475 | 0,75/0,4 | 4,5 | 475 | 0,5/0,4 | 10,5 |
| 16 | 416 | 0,9/0,5 | 3,5 | 755 | 0,6/0,5 | 11,8 | 422 | 0,5/0,4 | 2,3 | 471 | 0,75/0,4 | 12 |
| 17 | 403 | 0,75/0,4 | 5,6 | 546 | 0,5/0,4 | 22,4 | 521 | 0,75/0,4 | 5,6 | 603 | 0,75/0,4 | 22,4 |
| 18 | 456 | 0,75/0,4 | 1,1 | 551 | 0,75/0,4 | 7,1 | 456 | 0,5/0,4 | 1,9 | 475 | 0,5/0,4 | 7,8 |
| 19 | 420 | 1,0/0,5 | 2 | 951 | 1,2/0,5 | 27,7 | 365 | 1,4/0,5 | NA | 724 | 1,5/0,5 | 21,9 |

**Supplementary Table 1: pacing parameters of PCIS case.**

PCIS: Post cardiac injury syndrome, NA: not available.
